# Dataset of relationships among social media marketing activities, brand loyalty, revisit intention. Evidence from the hospitality industry in Northern Cyprus

**DOI:** 10.1016/j.dib.2018.11.024

**Published:** 2018-11-09

**Authors:** Blend Ibrahim, Ahmad Aljarah

**Affiliations:** a Girne American University, Business Management Department, via Mersin 10, North Cyprus, Turkey; b Girne American University, Marketing Department, via Mersin 10, North Cyprus, Turkey

## Abstract

The central purpose of this data article is to empirically investigate the relationships among social media marketing (SMM) activities, brand loyalty and revisit intention in five-star hotels in Northern Cyprus. Few researchers have investigated SMM activities, while none has looked at how SMM activities can be used toward improving brand loyalty and revisit intention in the tourism service industry. Hence, data gathered for the purposes of this research add to our understanding of today׳s social media marketing as a new generation marketing tool. This data was generated via a structured questionnaire, a total of 389 customers were surveyed who used five (5) hotels Facebook profiles, the hotels were all five-star ranked and located in Kyrenia city (Northern Cyprus). The data were examined by Structural Equation Modelling (SEM). Several analysis techniques have been used, the result showed a significant influence of SMM activities on brand loyalty and revisit intention, also the mediation outcome of brand trust is partially supported. Thus, consequential recommendations have been put forward.

**Specifications table**

**Table:** 

*Subject area*	*Business Management -Marketing – marketing communication*
*More specific subject area*	*Social media marketing (SMM) activities –Online social media-brand loyalty-revisit intention*
*Type of data*	*Table and figure*
*How data was acquired*	*Experiment*
*Data format*	*Raw data, analyzed statistical data*
*Experimental factors*	*Samples consist of five-star hotels customer in Northern Cyprus and interested in social media platforms (hotel Facebook page)*
*Experimental features*	*The social media marketing activities is manipulated; brand loyalty is measured though a four-item scales reflecting the behavioral and attitudinal loyalty; revisit intention is measured through a four-item scales.*
*Data source location*	*Kyrenia city, Northern Cyprus*
*Data accessibility*	*Data is contained in this article*
Related research article	

**Value of the data**

• This data article reports the role of social media marketing activities in enhancing brand loyalty and revisit intention in the hospitality industry by considering brand trust for hotel Facebook pages.
• The dataset describes the knowledge gap by developing a dataset model to examine the growing position of SMM. It similarly offers a model for marketers interested in predicting brand loyalty and revisit intention.
• The results acquired from the dataset showed a positive relationship between SMM activities and brand loyalty, revisit intention in the five-star hotel in Northern Cyprus.
• The dataset can be developed in the future in new data article or new research article – it can be extended to include new comparative study to explore social media platforms difference (i.e. Facebook, Instagram, Twitter), contexts (i.e. banks, sports, governmental), countries (i.e. developed, emerging, developing), demographic differences, international differences, culture differences (i.e. collectivism versus individualism).
• For researchers interested in social media we present a dataset that is the first to examine SMM activities role in predicting brand loyalty and revisit intentions while accounting for the effect of brand trust.

## 1 Data

The data produced here resulted from surveying SMM activities on brand loyalty and revisit intention while considering the mediating role of brand trust at a five-star hotel in Northern Cyprus through employing a 5-Likert scale. The social media marketing activities in our study context refer to a new framework that has already been developed by previous scholars [1], [2]. This framework evolves around five activities (entertainment, interaction, trendiness, customization and word of mouth (WOM)) that were used to investigate the role of SMM activities in customer equity and purchase intention in fashion brands. We extend on this previous work by studying the interaction between brands and customers as they play in a service industry. In order to test for the influence and strength of the relationships among the constructs of data article, the IBM SPSS AMOS program, (version22) is used to examine the dataset.

## 2 Experimental design, materials, and methods

The dataset presented a quantitative study based on experiment design. The data article examined the hospitality service industry focusing on five-star hotels in Kyrenia city in Northern Cyprus. The total population of five stars hotels customers derived is 789,903 tourists in 2017 [3]. The data sample was drawn from hotel customers of selected five (5) hotels in Kyrenia city from the list of 19 five stars hotels in northern Cyprus [3]**,** the five hotels in this data article selection was based those with the biggest bed capacity hotels in Kyrenia city The data sample was drawn from hotel customers of selected five (5) hotels in Kyrenia city from the list of 19 five stars hotels in northern Cyprus [3]**,** the five hotels in this data article selection was based those with the biggest bed capacity hotels in Kyrenia city with minimum 500 beds. The number of valid responses was 389.

**Fig. 1:**
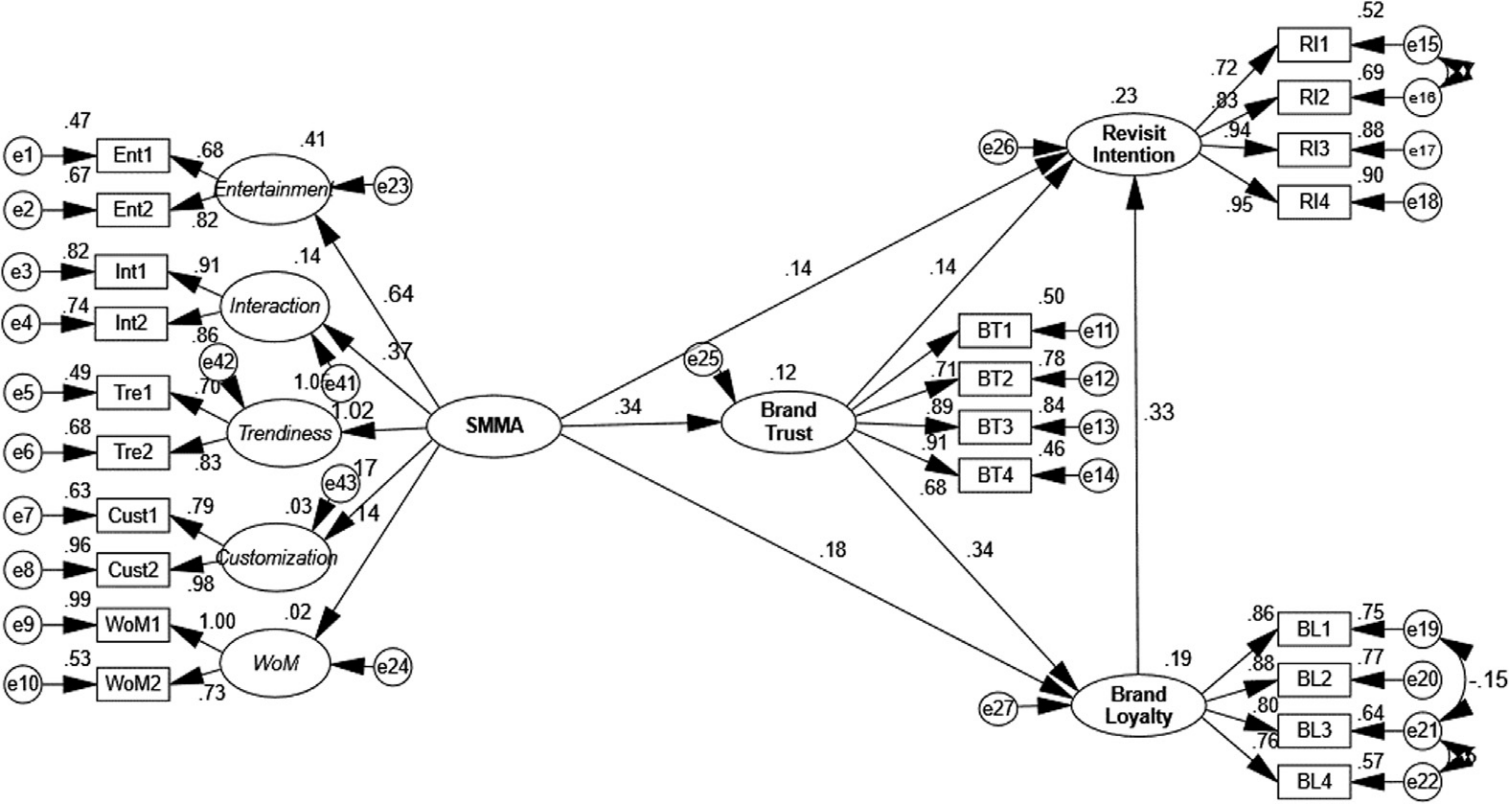
The structural Equation model for data set.

The authors used Confirmatory factor analysis (CFA) and goodness of fit indices to examine the validity of the measurement model. Several model indices were tested namely: (*x*^2^) measure, goodness-of-fit index (GFI), comparative fit index (CFI), Normed fit index (NFI), adjusted goodness of fit (AGFI) and the root mean square error of approximation (RMSEA). All model fit indices match with cut-off values depend on recommendations commonly used in literature [4], so the measurement model of dataset has acceptable where x^2^ = 2.20 < 3, CFI = 0.92 > 0.90, NFI = 0.92 > 80, CFI = 0.95 > 80,AGFI = 0.92 > 80, RMSEA = 0.05 < 0.08, and PCLOSE = 0.10 > 0.05. Finally, Fig. 1 shows the structural Equation model results for the dataset model (Table 1).

**Table 1:** Confirmatory factor analysis (CFA) and Goodness of fit indices.

**Goodness of fit indices**	**Index**		**Cut-off criteria**
	Before	After modification	
CMIN^2^/df	2.72	2.20	≤3
Goodness of fit (GFI)	0.90	0.92	>0.90
Normed fit index (NFI)	0.90	0.92	>0.90
Comparative fit index (CFI)	0.93	0.95	>0.90
Adjusted goodness of fit (AGFI)	0.86	0.89	>0.80
RMSEA	0.06	0.056	<0.08
PCLOSE	0.00	0.10	>0.05

Note: Cut-off criteria adopted from [4].

R.*χ*2 = CMIN/df.

The discriminant validity has been tested by adhering to tested recommendations [5]. The results for examining discriminant validity are shown in Table 2. The square root of the average variance extracted (AVE) for each construct is more than the correlations between this construct and any other construct. Also, AVE value should be greater than 0.50 which mentions the presence of an appropriate level of discriminate validity.

**Table 2:** Assessing discriminant validity.

	CR	AVE	MaxR(H)	BT	Int	Tre	Cus	Ent	BL	RI	WoM
Brand Trust (BT)	0.87	0.64	0.91	**0.80**							
Interaction (Int)	0.87	0.78	0.94	0.26	**0.88**						
Trendiness(Tre)	0.73	0.58	0.95	0.35	0.36	**0.76**					
Customization(Cus)	0.88	0.79	0.97	0.13	0.10	0.11	**0.88**				
Entertainment(Ent)	0.72	0.56	0.97	0.06	0.18	0.67	0.27	**0.75**			
Brand Loyalty(BL)	0.89	0.67	0.98	0.40	0.23	0.30	0.18	0.10	**0.82**		
Revisit Intention(RI)	0.91	0.73	0.98	0.31	0.23	0.28	0.46	0.08	0.42	**0.85**	
WoM	0.76	0.64	0.99	0.01	0.02	0.09	-0.04	0.04	0.02	-0.05	**0.80**

Table 3 shows the summary of the measurement model and all factors and items. Standardized loadings are above 0.50 and accepted. For reliability analysis, Cronbach׳s alpha is used and values ranged from 0.71 to 0.92 above the cutoff point 0.70 which considered acceptable [6]. The values of composite reliably (CR) scores are from 0.72 to 0.89, which is above 0.70 recommendations in the literature [7]. Similarly, the AVE values should be greater than 0.50 [5]. So, the values produced in our analysis have provided an overall indication of the convergent and discriminant validity of the measurement model.

**Table 3:** Summary of the measurement model.

Latent constructs	Item	Mean	SD	Loading	Cronbach׳s	CR	AVE
**Social media marketing activities**							
Entertainment					0.71	0.72	0.56
	ENT1	2.69	0.994	0.697			
	ENT2	3.27	0.915	0.808			
Interaction					0.84	0.87	0.78
	INT 1	2.25	0.911	0.912			
	INT 2	2.24	0.854	0.854			
Trendiness					0.73	0.73	0.58
	TRE 1	2.65	0.971	0.699			
	TRE2	3.23	0.959	0.824			
Customization					0.87	0.88	0.79
	CUS 1	4.05	0.716	0.809			
	CUS 2	4.03	0.717	0.963			
Word of mouth					0.84	0.76	0.64
	WoM1	3.03	1.368	1.322			
	WoM2	3.43	1.352	0.551			
**Brand Trust**					0.84	0.87	0.64
	BT 1	3.08	0.915	0.711			
	BT 2	2.99	0.943	0.886			
	BT 3	3.01	0.950	0.914			
	BT 4	2.88	0.895	0.679			
**Revisit Intention**					0.92	0.73	0.98
	RI 1	3.99	0.737	0.803			
	RI 2	3.96	0.715	0.935			
	RI 3	3.95	0.732	0.834			
	RI 4	3.95	0.709	0.851			
**Brand Loyalty**					0.89	0.89	0.67
	BL 1	3.53	0.915	0.848			
	BL 2	3.72	0.826	0.891			
	BL 3	3.37	0.951	0.777			
	BL 4	3.63	0.856	0.757			

Accessible in Table 4 are the values of correlation, statistics means and standard deviations among study constructs of data article. Overall the study shows significant associations of the studied model.

**Table 4:** Means, standard deviations (SD), and correlations of study construct.

Constructs	Mean	SD	1	2	3	4
SMMA	3.08	0.51	1	0.269**	0.249**	0.271**
Brand Loyalty	3.56	0.77	0.269**	1	0.387**	0.379**
Brand Trust	2.98	0.78	0.249**	0.387**	1	0.310**
Revisit Intention	3.96	0.65	0.271**	0.379**	0.310**	1

** Correlations are significant at the 0.01 level.

Table 5 shows the Structural Equation Model (SEM) and Goodness of fit indices, after modifying the model we attained an acceptable model as shown by the values of Goodness of fit indices.

**Table 5:** Structural Equation Model (SEM) and Goodness of fit indices.

**Goodness of fit indices**	**Index**		**Cut-off criteria**
	Before	After	
CMIN^2^/df	3.04	2.64	≤3
Goodness of fit (GFI)	0.88	0.90	>0.90
Normed fit index (NFI)	0.88	0.90	>0.90
Comparative fit index (CFI)	0.92	0.93	>0.90
Adjusted goodness of fit (AGFI)	0.85	0.87	>0.80
RMSEA	0.07	0.06	<0.08
PCLOSE	0.00	0.00	>0.05

Final analysis step is produced in Table 6. In panel A, direct effects of studied constructs is provided. While Panel B shows Mediation effects, the results show the partial mediation effect observed in our study.

**Table 6:** Regression weight and critical ratio and mediation effects.

Panel A: Regression weight and critical ration						
***Exogenous constructs***	***Endogenous constructs***	***Beta***	***SE***	***CR***	***p-value***	***L***
SMMA	Brand Loyalty	0.185	0.159	2.75	0.00	Sig
SMMA	Revisit Intention	0.147	0.154	2.31	0.02	Sig
SMMA	Brand Trust	0.34	0.255	4.24	***	Sig
Brand Loyalty	Revisit Intention	0.335	0.061	5.58	***	Sig
Brand Trust	Revisit Intention	0.147	0.044	2.52	0.01	Sig
Brand Trust	Brand Loyalty	0.342	0.044	5.83	***	Sig
Panel B: Mediation effects						
***Relationship***		***Direct Effect***	***Indirect Effect***	***Indirect***		
SMMA → Brand Trust →Revisit Intention		0.24 (0.01)	0.08 (0.01)	Partial Mediation		Sig
SMMA → Brand Trust →Brand Loyalty		0.22(0.03)	0.11 (0.00)	Partial Mediation		Sig

***. *P*-value is significant at the 0.001 level. S.E = Standard error; CR = Critical ratio; L = Label
